# Endovascular Sharp Recanalization for Calcified Femoropopliteal Artery Occlusion

**DOI:** 10.1155/2012/516027

**Published:** 2012-09-02

**Authors:** Hsuan-Li Huang, Hsin-Hua Chou, Tien-Yu Wu

**Affiliations:** Section of Cardiology, Department of Medicine, Buddhist Tzu Chi General Hospital, Taipei Branch, 289 Jiang Kuo Road, Xindian City, Taipei 23142, Taiwan

## Abstract

Endovascular intervention of peripheral chronic total occlusion (CTO) is technically challenging and time consuming. Various techniques and devices are used to facilitate lesion crossing and improve the success rate of the procedure. However, these new devices are quite expensive and not readily available. We report 2 cases of peripheral CTO wherein the occlusions were successfully crossed by using stiff end of Terumo glidewire. This sharp recanalization may be a useful technique for the recanalization of calcified peripheral CTOs when conventional techniques fail and new devices are not readily available, but it is accompanied by the risk of distal atheroembolism.

## 1. Introduction

Endovascular intervention (EVI) for the recanalization of peripheral chronic total occlusion (CTO) is technically challenging and time consuming. Patients with peripheral CTOs usually present with critical limb ischemia or disabling claudication [1]. Recanalization attempts fail in approximately 20% of the cases of peripheral CTOs because of difficulty in safely and reliably crossing heavily calcified lesions by using the standard guidewire and catheter techniques [2, 3]. Various techniques and devices have been developed to facilitate the introduction and placement of the guidewire into distal blood vessels. However, these devices are quite expensive and may not be readily available for use in routine clinical practice [4–9]. Very few reports have been published on the management of peripheral CTOs by using the sharp recanalization technique in which the stiff end of the glidewire is advanced into the artery. We report 2 cases of peripheral CTO wherein the occlusions were successfully crossed by the sharp recanalization technique after the conventional approach failed.

## 2. Case Reports

### 2.1. Case 1

**Figure 1:**
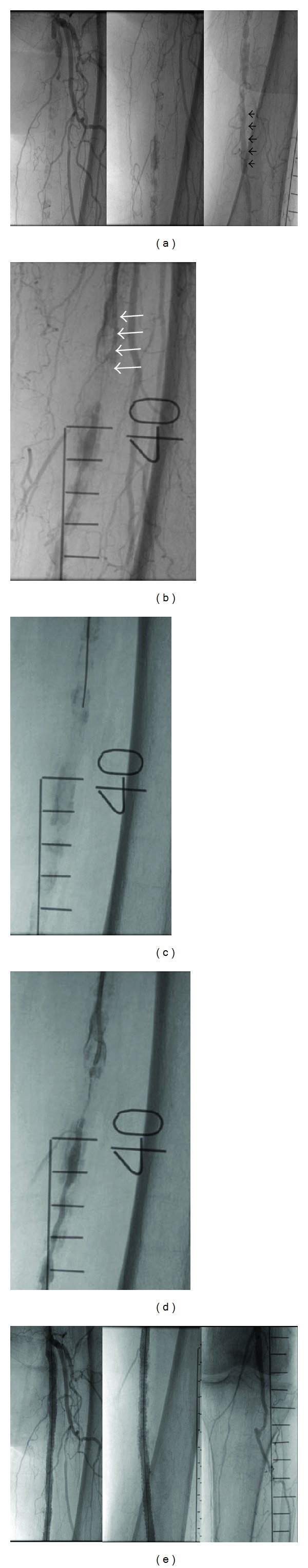
(a) Total occlusion of the superficial femoral artery (SFA) from the ostium onwards, heavily calcified plaques, and filling of the middle and distal portions of the SFA by collaterals is shown (black arrows). (b) A huge calcified plaque (white arrows) is noted in the proximal portion of the SFA, and this plaque impedes lesion crossing. (c) The stiff end of the Terumo glidewire is used for sharp recanalization. (d) The middle portion of the SFA was observed using a contrast medium that was injected through the right Judkin catheter after sharp recanalization. (e) Stent implantation from the proximal to the distal portion of the SFA is successful, and good angiographic results are noted.

A 67-year-old woman with a medical history of hypertension, end-stage renal disease, and left femoropopliteal artery bypass that was performed 3 years ago was referred to our institute for EVI because of gangrene of the left second toe and intractable pain. The ankle brachial index (ABI) was low at 0.35. The extremity of angiography revealed occlusion of the left superficial femoral artery (SFA) from its ostium with collaterals supplying the distal portion of the SFA and tibial arteries (Figure 1(a)). EVI was performed via the ipsilateral antegrade femoral approach. A 6-Fr introducer sheath (Radifocus Introducer II, Terumo, Tokyo, Japan) was placed in the artery after successful antegrade arterial puncture. Lesion crossing was unsuccessful when conventional techniques were applied using 0.014-in Conquest pro CTO guidewire (Asahi Intec, Aichi, Japan), 0.018-in V18-control wire (Boston Scientific, Natick, MA, USA), or 0.035-in Terumo stiff glidewire (Radifocus, Terumo, Tokyo, Japan) along with the balloon or exchange catheter. This is because a calcified hard plaque present in the proximal-middle region of the SFA blocked the reentry into the true lumen during subintimal angioplasty (Figure 1(b)). The plaque was carefully probed and penetrated using the stiff end of the Terumo glidewire that was supported by a 5-Fr right Judkins (JR) catheter (Figure 1(c)). The occluded lumen of the arterial portion distal to this plaque was visualized by using a contrast medium that was injected via the JR catheter (Figure 1(d)). A 300 cm long PT2 guidewire (Boston Scientific) was used to cross the lesion, and subsequently, angioplasty was performed. Two long Edwards Self-Expanding Lifestents (Bard, Edwards Lifesciences, Irvine, CA, USA) were deployed consecutively from the proximal to the distal portion of the SFA, and good angiographic results were obtained (Figure 1(e)). After the intervention, the ischemic symptoms resolved, and the ABI increased to 0.78. The patient's second toe was amputated 1 month after the intervention. Repeat intervention was performed 7 months after the index procedure because an additional lesion in the left common iliac artery and diffuse in-stent restenosis at the site of stent placement in the SFA were noted. The patient's condition was stable after the second intervention, and ischemic symptoms had disappeared.

### 2.2. Case 2

**Figure 2:**
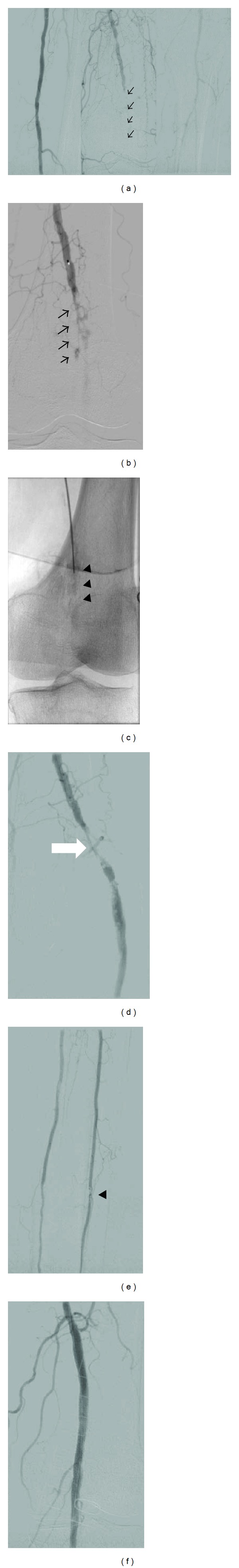
(a) Control angiography reveals a mild plaque in the proximal-middle region of the SFA and a short calcified occlusion in the proximal popliteal artery; distal popliteal artery is observed via few collaterals. (b) Lesion crossing results in subintimal wire tracking and development of the false lumen (black arrows). (c) The calcified occlusion was carefully probed (black arrowheads) by using the stiff end of the Terumo glidewire for sharp recanalization. (d) The true lumen is observed after sharp recanalization (white arrow). (e) Small atherosclerotic emboli are noted in the middle portion of peroneal artery (black arrowhead). (f) Good angiographic results are obtained after subsequent angioplasty and stent implantation.

An 82-year-old woman visited our hospital with critical left limb ischemia and impending foot cyanosis. The patient had a history of hypertension, diabetes mellitus, and coronary bypass surgery and had undergone SFA stenting and below-the-knee (BTK) angioplasty for the right leg in 2009. The ABI of the left leg was 0.45 at the time of admission, and the diagnostic angiography revealed heavily calcified occlusions in the proximal popliteal artery and very slow blood flow to the BTK area (Figure 2(a)). The antegrade flow was further compromised because of unsuccessful wire crossing and subintimal tracking in the region of the calcified plaque (Figure 2(b)). The stiff end of the Terumo glidewire was used to carefully probe this plaque (Figure 2(c)), after which the true lumen could be observed (Figure 2(d)). A 300 cm long PT2 guidewire was used to cross the lesion, and thereafter, balloon angioplasty was performed. Small atherosclerotic emboli were noted in the middle region of the peroneal artery (Figure 2(e)) after sharp recanalization. These emboli were successfully suctioned out by using a thrombus-aspiration catheter (Thrombuster, Kaneka, Kanagawa, Japan). A Lifestent (Bard, Edwards Lifesciences) was positioned in the proximal popliteal artery, and a good angiographic result without evidence of distal embolism was obtained after the intervention (Figure 2(f)). The ABI measured 1 week after EVI increased to 0.94, and ischemic symptoms were absent. No other symptoms were noted until 3 months after the intervention.

## 3. Discussion

Percutaneous endovascular techniques have revolutionized the management of symptomatic peripheral arterial occlusive disease (PAOD) when conservative medical treatment fails; patients who undergo treatment using these techniques have lower morbidity and recover earlier than patients treated by surgical bypass [2]. Peripheral CTOs commonly occur in patients with limb ischemia, and recanalization of these occlusions is technically challenging and time-consuming. Short, noncalcified lesions without flush occlusion are easily treated by intraluminally crossing the occlusion by using a guidewire. However, when the fibrous occlusion is long, densely organized, and homogenous, subintimal angioplasty is the widely accepted modality for treating this occlusion, and the success rate of subintimal angioplasty is higher (70–90%) than that of treatment involving intentional true lumen passage [9]. However, successful reentry by the subintimal approach is difficult if the patient has densely calcified lesions, diffuse disease of the distal target, or small caliber lumens, as described in Case 1. New devices and techniques are used for facilitating the recanalization of peripheral CTOs, including reentry catheters [5, 6], blunt microdissection catheters [7], optical coherence reflectometry, excimer laser, and ultrasound-guided vibration angioplasty [8]. Although effective, these devices are quite expensive and not readily available. The sharp recanalization technique using a needle or needle-tipped guidewire has been used for the treatment of central vein occlusion [10] or the fenestration of aortic dissection [11]. However, few reports have been published regarding the crossing of peripheral CTOs by using the stiff end of the Terumo glidewire. We speculated that the stiff end of the glidewire would provide greater penetration power for sharp recanalization than that available with the use of the conventional wire tip; the stiff end can be used to weaken and create channels in calcified hard plaques, which may facilitate occlusion crossing. Certainly, the use of the stiff end of the glidewire increases the risk of vessel perforation. To avoid this complication, careful manipulation of the glidewire using a fluoroscope is warranted, and the glidewire should be replaced with a soft-tip wire as soon as the total occlusion is crossed. Kjellgren et al. reported successful crossing of the peripheral CTO without complications when the stiff backend of the glidewire was used [12]. However, atheroembolism may occur during sharp recanalization, as described in Case 2. 

 In this report, we describe the use of the stiff end of the glidewire in facilitating intraluminal crossing of highly resistant calcified peripheral CTOs when conventional techniques fail or new devices are not available. We have found that sharp recanalization by cautious use of the stiff end of the glidewire can facilitate lesion crossing and be a useful technique for the management of peripheral CTOs; however, the possibility of distal atheroembolism should be considered.
